# The role of circadian rhythm in osteoporosis; a review

**DOI:** 10.3389/fcell.2022.960456

**Published:** 2022-09-27

**Authors:** Yihao Tian, Jian Ming

**Affiliations:** Department of Pathology, General Hospital of Northern Theater Command, Shenyang, China

**Keywords:** circadian rhythm, melatonin, osteoclast differentiation, circadian rhythm genes, osteoporosis

## Abstract

Osteoporosis is characterized by a high incidence rate, with significant effects on people’s lives. The underlying mechanisms are complex, with no treatments for the condition. Recent studies have indicated that melatonin can be used to treat osteoporosis by promoting osteoblast proliferation and differentiation, and inhibiting osteoclast differentiation. Specifically, *in vivo* mechanisms are initiated by stabilizing biological rhythms in bone tissue. In healthy organisms, these biological rhythms are present in bone tissue, and are characterized by bone formation during the day, and bone resorption at night. When this rhythm is disrupted, osteoporosis occurs. Thus, taking appropriate medication at different times of the day could produce different effects on osteoporosis rhythms. In this review, we characterized these processes, and provided treatments and management strategies for individuals with osteoporosis.

## 1 Osteoporosis

**FIGURE 1:**
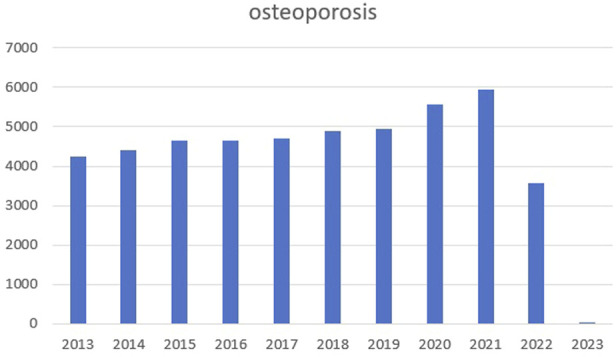
The number of articles in the past 10 years with osteoporosis.

Osteoporosis is a complex pathogenic metabolic disease (Loffler et al., 2020; Zhang et al., 2022) caused by imbalanced bone formation and resorption (Johnston & Slemenda, 1995; Gu et al., 2020). By 2050, the number of hip fracture patients in Asia will more than double, accounting for 50% of the total hip fracture in the world (Wu et al., 2021). We searched PubMed for relevant articles in the past 10 years with osteoporosis as the keyword and made statistics. We found that the number of articles in the past two or 3 years has increased significantly, as shown in the figure below (Figure 1). The Wnt signaling pathway is believed to play an important role in bone homeostasis and bone related diseases, hence new osteoporosis therapies targeting Wnt signaling are emerging (Manolagas, 2014; Zhang et al., 2022). Similarly, the protein kinase B (Akt) signaling pathway is closely associated with osteoporosis treatments (Liu et al., 2018; Jiang et al., 2022). In ovariectomized rats, the Mongolian medicine, echinops has been shown to be effective for osteoporosis by Akt (Liu et al., 2018). In an osteoporosis rat model, mitogen activated protein kinase (MAPK) is involved in osteoblast proliferation and differentiation (Pan et al., 2018). While in RAW264.7 cells, bisphosphonate zoledronate inhibits osteoclast migration, differentiation and bone resorption *via* the AMP-activated protein kinase (AMPK) pathway, suggesting this route could become a new target for disease treatment (Dong et al., 2018). Although Wnt/Akt/MAPK/AMPK pathways exert important functions in bone homeostasis and bone related diseases, and new therapies are constantly emerging, the current therapeutic environment for osteoporosis is limited, therefore new side-effect free drugs and therapeutic targets are required (Gera et al., 2022; Kobayakawa et al., 2022).

## 2 Melatonin and diabetic osteoporosis

Melatonin is a hormone secreted by the pineal gland, and its chemical essence is a methoxyindole (Amaral & Cipolla-Neto, 2018). Melatonin secretion generates a typical circadian rhythm, which helps regulating the normal circadian rhythms of the body (Amaral & Cipolla-Neto, 2018). Recent studies have shown the hormone acts on bone tissue to exert its biological effects. Low melatonin concentrations have been used to treat osteoporosis by promoting bone growth (Xiao et al., 2020), while high concentrations treat scoliosis by inhibiting this growth (Qiu et al., 2020). Melatonin improved bone trabecular microstructure and reduced autophagy levels (50 mg/kg was more efficacious than 100 mg/kg) in 4-month-old male SPF SD rats (Zhang et al., 2016). In osteoblasts cultured in high glucose, melatonin promoted osteogenic activities, and appeared to inhibit autophagy levels by inhibiting the extracellular-regulated protein kinase (ERK) signaling pathway (10 μM was better than 1 mM), and delayed diabetic osteoporosis (Zhang et al., 2016). In 6-week-old adult female SD rats, when compared with the diabetic alone group, body weight, serum superoxide dismutase (SOD), glutathione peroxidase (GSH-Px), and Ca^2+^ levels, lumbar spine and left and right femur BMD of the diabetic + melatonin group were significantly increased), while blood glucose, serum malondialdehyde (MDA), and parathyroid hormone (PTH) levels were significantly decreased and no significant differences in serum phosphorus levels (Jing & Wang, 2017). Aerobic exercise and melatonin improve diabetic osteoporosis; both are more effective than exercise alone (Jing & Wang, 2017). Thus, melatonin may effectively reduce blood Ca^2+^ and PTH levels, augment BMD by improving antioxidant stress, reducing the effects of high glucose oxidative stress on bone metabolism, and regulating glucose metabolism (Jing & Wang, 2017).

## 3 Melatonin and osteoclast differentiation

### 3.1 Signaling pathways

**FIGURE 2:**
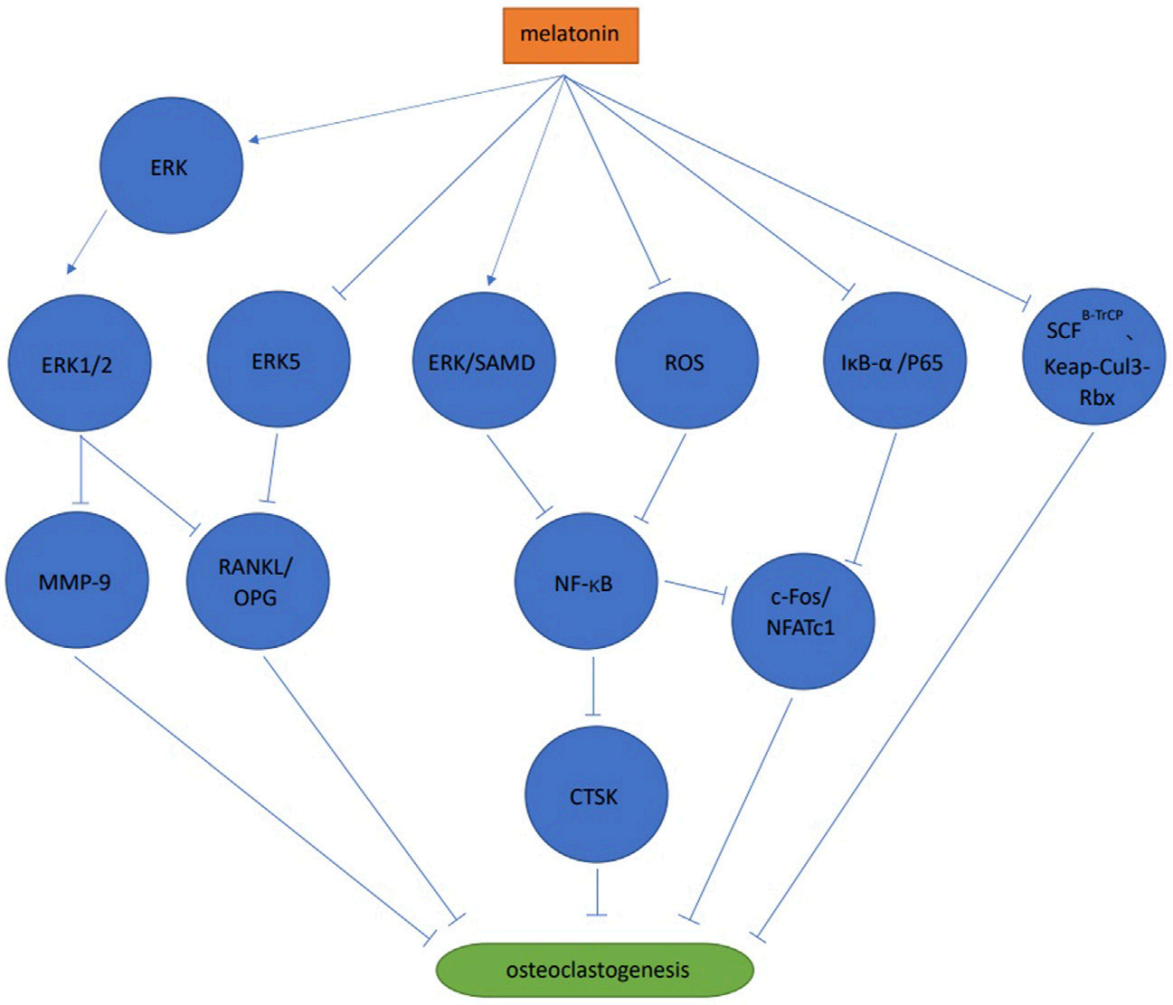
Signaling pathways of melatonin and osteoclast differentiation.

Melatonin activated the ERK1/2 signaling pathway by phosphorylating ERK in RAW264.7 cells, thereby inhibiting osteoclast differentiation *via* the melatonin receptor (Satue et al., 2015). When compared with the control group, melatonin significantly inhibited bone resorption, but no significant differences were observed in tartrate-resistant acid phosphatase (TRAP) positive cell numbers (Satue et al., 2015). Melatonin also significantly reduced matrix metallopeptidase nine expression, but no significant effects on cathepsin K expression were observed (Satue et al., 2015). Melatonin inhibited osteoclast differentiation and cathepsin K expression *via* ERK and NF-κB pathways in RAW264.7 cells (Wang et al., 2019). Also in RAW264.7 cells, melatonin significantly inhibited NFATc1 and c-Fos expression by blocking the phosphorylation of IjB-A and p65 rather than IKKa, to inhibit receptor activator of nuclear factor-kappa B ligand (RANKL) induced osteoclast differentiation, F-actin loop formation and bone resorption, in a melatonin concentration dependent manner. The study also reported that melatonin exerted no effects on mitogen-activated protein kinases (MAPK) and phosphoinositide 3-kinase/protein kinase B (PI3K/AKT) signaling pathways (Ping et al., 2017b). When co-culturing human mesenchymal stem cell (MSC) and peripheral blood mononuclear cell (PBMCs), MSDK (melatonin, strontium (citrate), vitamin D3 and vitamin K2) inhibited osteoclast differentiation by increasing osteoprotegerin (OPG) and reducing RANKL levels (Maria et al., 2017). These authors observed that melatonin increased OPG/RANKL levels *via* MEK1/2 and MEK5 signaling pathways to inhibit osteoclast differentiation. This process was mediated by the melatonin receptor type 1 B (MT2) (Maria et al., 2018). During osteoclast differentiation of mouse bone marrow mononuclear cells (BMMS) induced by RANKL, melatonin significantly inhibited osteoclast differentiation at pharmacological concentrations (10^−4^ mol/L, 10^−5^ mol/L, 10^−6^ mol/L) but not physiological concentrations (10^−8^ mol/L, 10^−9^ mol/L, 10^−10^ mol/L, 10^−11^ mol/L). The results showed that the process was melatonin concentration dependent. Melatonin inhibited osteoclast differentiation and bone resorption by inhibiting the NF-κB signaling pathway, mediated by reactive oxygen species but not SIRT1 (Zhou et al., 2017). In mouse BMMSs melatonin inhibited osteoclast differentiation by down-regulating the NF-κB pathway, thereby reducing NFATc1 expression. However, these anti-osteoclast differentiation effects were not associated with the melatonin receptor type 1A (MT1) and MT2, and no effects were observed towards the MAPKs (ERK, JNK, and p38) (Kim et al., 2017). Melatonin inhibited osteoclast differentiation *via* ubiquitination, which is a process mediated by ubiquitin ligase SCF^B−TrCP^ and Kelch-like ECH-associated protein 1-cullin 3-RING-box 1 (Keap-Cul3-Rbx), or the proteasome (Vriend & Reiter, 2016). Melatonin effectively inhibited osteoclast differentiation and bone resorption in mouse bone marrow cells (including osteoclast and osteoblast precursors) at pharmacological concentrations (1 × 10^−4^ mol/L–5 × 10^−4^ mol/L). This process may be mediated by other cells (e.g., bone marrow stem cells) rather than melatonin acting directly on osteoclasts, suggesting interactions between different osteocytes could regulate the bone resorption/formation balance (Koyama et al., 2002) (Figure 2).

### 3.2 Animal studies

**FIGURE 3:**
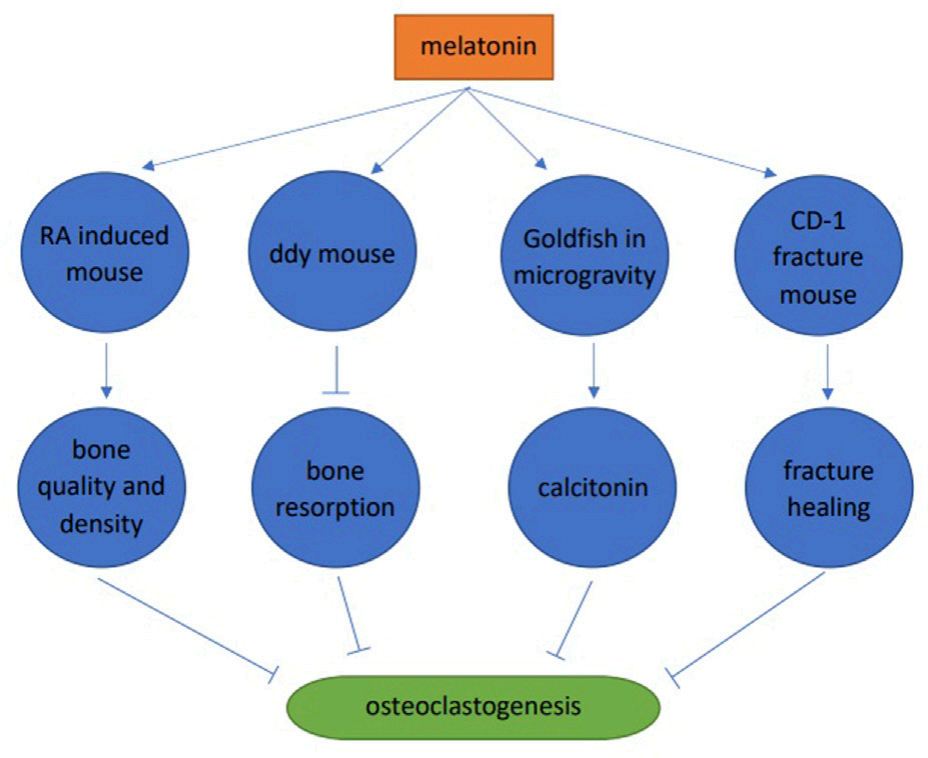
Animal studies of melatonin and osteoclast differentiation.

Melatonin significantly improved the microstructure of bone trabeculae, increased bone trabeculae in the retinoic acid-induced osteoporosis mouse model, improved femur and vertebrae microstructures, and improved bone quality and density (Wang et al., 2019). Melatonin (pharmacological dose) inhibited bone resorption and increased bone mass in 4-week-old male ddY mice. Melatonin significantly increased BMD (36%), bone mass (49%), and trabecular thickness (19%). These skeletal improvements were believed to be due to melatonin inhibiting RANKL expression and secretion in osteoblasts, and further inhibiting osteoclast differentiation (Koyama et al., 2002). Melatonin inhibited osteoclast differentiation in goldfish scales by promoting calcitonin secretion in microgravity environments in space (Ikegame et al., 2019). In a CD-1 fracture mouse model, melatonin promoted fracture healing by inhibiting RANKL induced osteoclast differentiation, thereby reducing bone resorption (Histing et al., 2012) (Figure 3).

### 3.3 Peri-prosthetic osteolysis

From radiological and histomorphological studies, melatonin inhibited bone resorption and promoted bone formation in a pericranial osteolysis mouse model, induced by titanium particles. When compared with untreated mice, numbers of osteoclasts in mice treated with low and high melatonin concentrations decreased significantly, with melatonin appearing to regulate RANKL/OPG levels, mediated by the Wnt/β-catenin signaling pathway (Ping et al., 2017a).

### 3.4 Human studies

**FIGURE 4:**
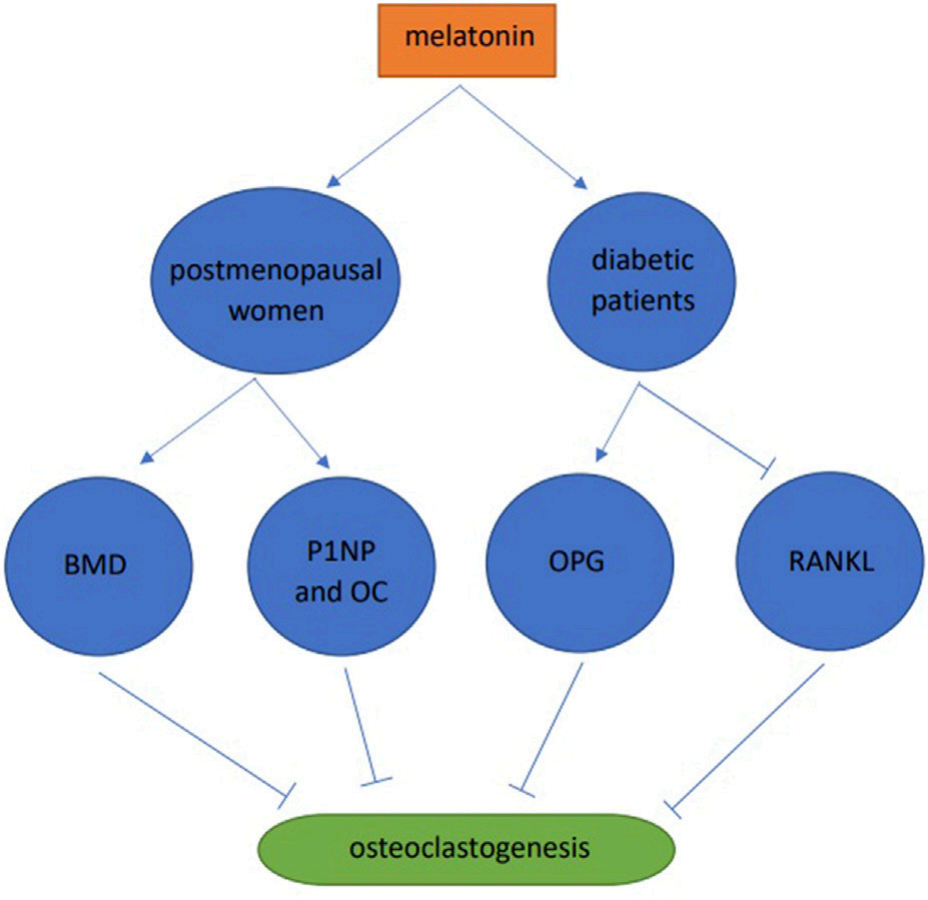
Human studies of melatonin and osteoclast differentiation.

A one-year double-blind clinical trial in postmenopausal women was conducted and when compared with the placebo group, the BMD of patients in the MSDK group increased by 4.3% and 2.2% in the lumbar spine and left hip, respectively. In addition, melatonin increased serum levels of the bone formation markers, one procollagen n-propyl peptide (P1NP) and osteocalcin (OC). At the same time, there are significant improvements of mood and sleep quality in MSDK group (Maria et al., 2017). 30 healthy and 30 diabetic subjects were studied, and when compared with the healthy group, RANKL levels in diabetic saliva were increased, OPG was decreased, and blood melatonin was decreased. After taking melatonin, the periodontal markers, gingival index and pocket depth were significantly increased, RANKL in saliva was significantly decreased, and OPG increased (Cutando et al., 2014). Combined, these data showed that melatonin slowed down osteoclast formation by improving alveolar bone quality and preventing periodontal disease (Figure 4).

## 4 Circadian rhythm genes

**FIGURE 5:**
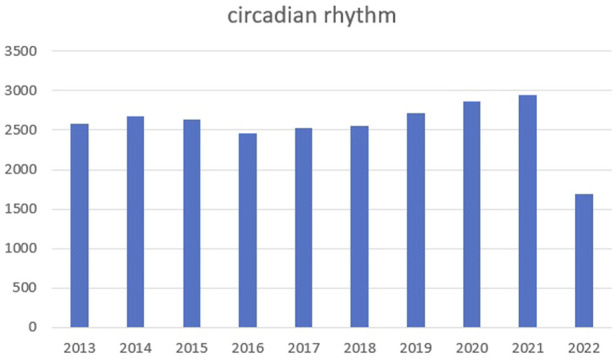
The number of articles in the past 10 years with circadian rhythm.

Circadian rhythm reflects alternations in daily biological behaviors and physiological activities, such as sleeping, waking, eating, and fasting (Welz et al., 2019; Stothard et al., 2020). We searched PubMed for relevant articles in the past 10 years with circadian rhythm as the keyword and made statistics. We found that the number of articles in the past two or 3 years has increased significantly, as shown in the figure below (Figure 5). *In vivo*, circadian rhythms are regulated and maintained by endogenous clock genes (Oishi et al., 2005; Liu et al., 2022), which are involved in several physiological activities, including metabolism (Peek et al., 2017), cell cycle (Han et al., 2016; Dong et al., 2019), sleep wake cycle (Ikegami et al., 2019), bone formation (Fu et al., 2005; Sato et al., 2007; Hirata et al., 2022; Zhao et al., 2022), and heart rate and blood pressure (Wang et al., 2008). However, when clock gene expression is aberrant, the body experience abnormalities and possible disease, including sleep disorders (Patke et al., 2017), diabetes (Marcheva et al., 2010), obesity (Paschos et al., 2012; Chaix et al., 2019), osteoporosis (Tang et al., 2020), and tumors (Altman et al., 2015). Thus, for disease diagnostics and treatments, the significance of body rhythms is vital. For example, when doing blood test, we recommend at the same time of the day. What is more, taking medicine at different times of the day can achieve different effects.

In mammals, the suprachiasmatic nucleus of the hypothalamus regulates circadian rhythms, with light driving the process through the retinal hypothalamic tract (Crosio et al., 2000). A series of complex protein networks produce stable 24-h circadian rhythms (Edery et al., 1994). Of these, heterodimeric CLOCK-BMAL1 acts on promoter or intronic sequences of PER and CRY genes to activate transcription and translation *via* the E-box enhancer, thus exerting positive regulatory roles (Hamaguchi et al., 2004; Kavakli et al., 2022). The PER and CRY complex enter the nucleus from the cytoplasm and inhibits the transcription and translation of CLOCK-BMAL1, thus exerting a negative regulatory role (Hirayama et al., 2003; Rasmussen et al., 2022). After CLOCK-BMAL1 levels are reduced, PER and CRY transcription and translation decreases, and CLOCK-BMAL1 inhibition decreases, thus forming a feedback loop (Liu et al., 2008).

ROR and REV-ERB are important regulators of this feedback loop (Liu et al., 2008; Gustafson & Partch, 2015; Mansingh & Handschin, 2022). The transcription and translation of REV-ERB is activated by the CLOCK-BMAL1 heterodimer and inhibited by CRY/PER, resulting in circadian rhythm changes in REV-ERBα (Liu et al., 2008; Gustafson & Partch, 2015). Conversely, REV-ERBα inhibits the transcription and translation of BMAL1 and CLOCK. RORα promotes the transcription and translation of BMAL1 and CLOCK by binding to the DNA binding element rore in the BMAL1 promoter competitively with REV-ERBα(Guillaumond et al., 2005). The REV-ERBα/RORα feedback loop and the positive and negative limbs of the circadian clock gene loop interact to maintain circadian rhythm stability.

## 5 Biorhythms and bone metabolism during the normal physiological state

### 5.1 *In vitro* bone remodeling markers are rhythmic in nature

The Per1 luciferase transgene was used as a bioluminescent indicator in the calvaria of neonatal rats *in vitro*, and the Per1 rhythmic expression cycle was 25 ± 4 h (McElderry et al., 2013). The tibial cortical bone of 8-week-old rats was resected, cultured *in vitro*, and bone collagen formation exhibited circadian rhythm fluctuations. When compared with daytime light, bone collagen accumulation and secretion was less in dark periods (Russell et al., 1985) (Table 1).

**TABLE 1:** *In vitro* bone remodeling markers are rhythmic in nature.

Molecule	Rhythm	Reference
Per1	25±4	McElderry et al. (2013)
Bone collagen	Accumulation and secretion (Light)	Russell et al. (1985)

### 5.2 Animal bone remodeling markers are rhythmic in nature

Several studies have shown that *in vivo* bone tissue metabolism generates a typical circadian rhythm, i.e., bone formation is dominant during the day, while bone absorption is dominant at night. Specifically, bone remodeling markers show a circadian rhythm. A non-invasive Raman microscope was used to track mineral accumulation in mouse skull bone tissue, and the rhythmic cycle of mineral deposition was 26.8 ± 9.6 h, and the earliest mineralization events occurred 6 hours after peak expression levels of Per1 (McElderry et al., 2013). Healthy adult female camels were studied and serum osteocalcin (OC) had a circadian rhythm over 24 h/day, with the lowest level at 13:00, and the highest at 18:00. Bone alkaline phosphatase (BAP) levels fluctuated slightly, reaching the lowest and highest levels at 1:00 and 12:00 noon, respectively (Al-Sobayil, 2010). In a biopsy study, bone tissue mineralization in the metaphysis of 4-week-old rats followed a circadian rhythm, indicating that calcium and inorganic phosphorus levels peaked during dark periods (Russell et al., 1984). Luminous signal intensity of osteoblasts (the human OC promoter was tagged with a luciferase reporter) in transgenic mice was analyzed, and the mouse maxillary complex, skull, tail, carpal bone, and tarsal bone all displayed oscillation modes of osteogenic activity (Gafni et al., 2009). In their transcriptome analysis, 26% of genes displayed circadian rhythms after a 12 h light/12 h dark cycle treatment. Two circadian rhythm transcription complexes (BMAL1/CLOCK and CRY/PER) and their mediators (DBP and REV-ERBα) showed oscillatory expression profiles (Zvonic et al., 2007). The strongest bone remodeling occurred in the photoperiod (9:00–21:00) (Simmons & Nichols, 1966). 5-Week-old male rats were subjected to a 12 h light/12 h dark, and serum TRAP, pyridinol (PYD), OC, Ca^2+^, and Pi levels displayed significant circadian rhythms, with peak levels between 9:00 and 13:00, and lowest levels between 21:00 and 1:00. In addition, alkaline phosphatase (ALP) and CT serum levels showed peak levels between 1:00 and 5:00. However, the authors observed no circadian rhythm changes in PTH serum levels (Shao et al., 2003). Per2 fluorescent mice was used to culture long (proximal femur and radius) and flat bone (skull and scapula) tissue *in vitro* for 6 months. Articular and epiphyseal cartilage in the growth plates of young mice underwent circadian rhythm, indicating peripheral bone tissue underwent circadian rhythm, independent of the central nervous system (Okubo et al., 2013). 4-week-old male rats were studied and matrix synthesis and secretion activities of chondrocytes and osteoblasts at different osteogenic sites, experienced the same circadian rhythms, i.e., peak levels appeared in the13:00 photoperiod, and the lowest levels appeared in the 1:00 dark period (Igarashi et al., 2013). In 6-week-old C3H/HEJ (C3H) mice, OC and C-terminal peptide levels underwent diurnal changes, with peak levels between 9:00 and 12:00, and the lowest levels between 15:00 and 18:00; the peak level was 26%–66% higher than the 24-h average level. However, serum ALP levels were less affected by circadian rhythms (Srivastava et al., 2001). Fibroblast growth factor 23 (FGF23) regulates phosphorus levels in osteoblasts. FGF23 expression levels displayed circadian rhythms in mice, i.e., serum levels were low in the daytime, but increased at night (Kawai et al., 2014). In the UMR-106 mouse osteoblast cell line, sympathetic nerves activated the expression levels of FGF23, and CRY1 inhibited this process by inhibiting ISO induced cAMP response element-binding protein (CREB) phosphorylation. Also, in BMAL1 knockout mice, the expression level of CRY1 was increased, and above process was also inhibited. These data indicated that FGF23 regulation by the sympathetic nerve was mediated by rhythmic proteins (Kawai et al., 2014) (Table 2).

**TABLE 2:** Animal bone remodeling markers are rhythmic in nature.

Animal	Molecule/Biological activity	Rhythm	Reference
Mouse skull bone	Mineral deposition	26.8±9.6	McElderry et al. (2013)
Healthy adult female camels	OC	13:00-	Al-Sobayil. (2010)
		18:00+	
	BAP	01:00-	
		12:00+	
Rat tibia	Ca^2+^,Pi	Dark+	Russell et al. (1984)
Transgenic mice	Osteogenic activity	Oscillation modes	Gafni et al. (2009)
Mouse skull bone	BMAL1/CLOCK and CRY/PER	Oscillatory expression profiles	Zvonic et al. (2007)
Rat femur and tibia	Bone remodeling	09:00∼21:00	Simmons & Nichols. (1966)
Male rat	TRAP,PYD,OC,Ca^2+^,and Pi	09:00∼13:00+	Shao et al. (2003)
		21:00∼01:00-	
	ALP,CT	01:00∼05:00+	
Per2 fluorescent mice	Long and flat bone	Rhythm independent of the central nervous	Okubo et al. (2013)
Male rats	Matrix synthesis and the secretion activities	01:00-	Igarashi, (2013)
		13:00+	
C3H mice	OC and C-terminal peptide	19:00∼12:00+	Srivastava et al. (2001)
		15:00∼18:00	
Mice	FGF23	Dark+	Kawai et al. (2014)
		Light-	

-:nadir, +:zenith

### 5.3 Human bone remodeling markers are rhythmic in nature

Serum collagen type 1 C-telopeptide (CTX), FGF23, sclerostin (SOST), and P1NP levels in healthy men (20–65 years old) were measured, and the bone resorption marker, CTX, exhibited peak circadian rhythms at 5:30 (range 1:30–07:30), FGF23 exhibited uncertain peak rhythms (range 2:30–11:30), while SOST and PINP exhibited none (Swanson C. et al., 2017). 100 volunteers were studied and serum CTX levels exhibited rhythmic changes over 24 h, with the highest levels at around 5:00 in the morning, and the lowest levels around 14:00 in the afternoon. Except to fasting, this rhythmic change was not affected by gender, age, menstrual status, bed rest, and other factors (Qvist et al., 2002). Saliva, serum, and urine from perimenopausal healthy women were analyzed at 9:30, 13:30 and, 17:30 and CTX levels peaked in the morning and dipped in the afternoon (Pellegrini et al., 2012). Peripheral venous blood (within 24 h) was collected from elderly men and premenopausal and postmenopausal women every hour, and PTH, OPG, and CTX levels displayed circadian rhythms. The highest OPG levels appeared in the daytime and the lowest appeared at night, while PTH and CTX levels had opposite patterns (Joseph et al., 2007). 20 women (25–65 years old) were studied and serum OC and CTX had the lowest levels during the day, and the highest at night. However, similar rhythms for OPG and soluble RANKL (sRANKL) were not detected in circulating blood (Dovio et al., 2008). The blood from 15 young women was analyzed at 8:00, 14:00, and 20:00, and CTX levels were increased in the morning, but decreased in the afternoon and night. However, no similar rhythms for ALP, OPG, and sRANKL were detected in circulating blood (Shimizu et al., 2009). Blood from volunteers aged 60–75 years old, comprising Gambian, Chinese, and British adults was studied. While there were ethnic differences in plasma levels of bone metabolism and PTH markers, their circadian rhythms were similar, i.e., plasma levels of CTX, P1NP, OC, and BAP showed rhythmic changes (Redmond et al., 2016). In a study involving six healthy men and four healthy women (20–30 years old), serum OC levels were decreased in the morning, but gradually increased in the afternoon and evening, and peaked at night (Gundberg et al., 1985). 14 women (74 ± 6 years old) and 14 men (80 ± 5 years old) were studied, and serum OC, ALP, urinary N-terminal peptide cross-linked collagen type 1, serum calcium, and PTH all exhibited biological rhythms (Greenspan et al., 1997) (Table 3).

**TABLE 3:** Human bone remodeling markers are rhythmic in nature.

Human	Molecule	Rhythm	Reference
10 healthy men	CTX	05:30+	Swanson et al. (2017a)
100 volunteers	CTX	14:00-	Qvist et al. (2002)
		05:00+	
40 perimenopausal healthy woman	CTX	Morning+	Pellegrini et al. (2012)
		Afternoon-	
18 elderly men and premenopausal and postmenopausal women	OPG	Day+	Joseph et al. (2007)
		Night-	
	PTH,CTX	Day-	
		Night+	
20 women	OC,CTX	Day-	Dovio et al. (2008)
		Night+	
15 young women	CTX	Morning+	Shimizu et al. (2009)
		Afternoon-	
		Night-	
90 Gambian,Chinese,and British adults	CTX,P1NP,OC, and BAP	Rhythmic changes	Redmond et al. (2016)
6 healthy men and 4 healthy women	OC	Day-	Gundberg et al. (1985)
		Night+	
14 women and 14 men	OC,ALP,NTX,Ca^2+^ and PTH	Biological rhythms	Greenspan et al. (1997)

-:nadir, +:zenith

## 6 Biological rhythm disorders in bone tissue could lead to osteoporosis

The 24-h circadian rhythm is based on light/dark alternations, and facilitates sleep behaviors at night and awake activities during the day. Jet lag, fasting, eating, etc., disturb these rhythms, resulting in osteoporosis.

### 6.1 Cell/gene knockout models

Cry and Per genes in mouse osteoblasts were knocked out and the bone mass of vertebrae and long bones increased significantly (Fu et al., 2005). RANKL expression and osteoclasts increased in osteoblast specific BMAL1 knockout mice, while in BMAL1 overexpression mice, both RANKL and osteoclast levels decreased. These observations indicated that bone resorption and bone mass were regulated by the clock gene, BMAL1, in osteoblasts (Takarada et al., 2017). After a BMAL1 specific knockout mouse, osteoclast differentiation decreased and bone mass increased, suggesting BMAL1 up-regulated NFATc1 expression by binding to the E-box element of the NFATc1 promoter (Xu et al., 2016). Mouse per2 and cry2 genes were knocked out, and there are different pathways to regulate bone remodeling between per2 and cry2. Cry2 mainly affected the biological process of osteoclasts, and the content of TRAP in serum is significantly reduced after knockout. Per2 acts on the biological process related to osteoblasts, and the bone formation rate (BFR) is significantly increased after knockout (Maronde et al., 2010). In BMAL1 gene knockout mice, when compared with wild-type animals, the bone bridge between metaphysis and epiphysis in knockout mice had shortened long bones, thinned bone cortex, thinned bone trabecula, reduced bone density, accelerated aging, and decreased bone marrow MSC differentiation into osteoblasts (Samsa et al., 2016). BMD and volume of bone were decreased in BMAL1 knockout mice when compared with wild-type mice (Kondratov et al., 2006). BMAL1 restored osteogenic abilities by inhibiting the NF-κB signaling pathway during inhibition of MSC differentiation, induced by type 2 diabetes, and regulated the balance between osteogenesis and osteoclast differentiation (Li et al., 2018). MSCs were cultured in osteogenic induction medium, and REV-ERBα expression decreased during bone MSC osteogenesis, while REV-ERBα overexpression inhibited bone MSC proliferation and osteogenic ability (He et al., 2015) (Table 4).

**TABLE 4:** Biological rhythm disorders in bone tissue lead to osteoporosis in cell/gene knockout models.

Animal/Cell	Interference	Consequence		Reference
Mouse osteoblast	Cry and Per-	Bone mass	↑	Fu et al. (2005)
Mouse osteoblast	Bmall-	RANKL and osteoclasts	↑	Takarada et al. (2017)
	Bmall+	RANKL and osteoclasts	↓	
Mouse osteoclasts	Bmall-	Osteoclasts	↓	Xu et al. (2016)
		Bone mass	↑	
Mice	Cry2-	Trap	↓	Maronde et al. (2010)
	Per2-	BFR	↑	
Mice	Bmall-	Bone cortex, bone trabecula, and bone density	↓	Samsa et al. (2016)
Mice	Bmall-	BMD and volume of bone	↓	Kondratov et al. (2016)
MSC	Bmal+	Osteogenic abilities	↑	Li et al. (2018)
MSC	REV-ERBα+	Osteogenic abilities	↓	He et al. (2015)

-:Knock out, +:overexpression, ↑:increase, ↓:decrease

### 6.2 Animal studies

Wild-type mice were exposed to a 24 h light for 24 weeks. Their data showed that the central circadian rhythm pacing rate of suprachiasmatic nucleus (SCN) neurons decreased by 70%, with trabecular density also decreased. When mice returned to standard light/dark cycles, SCN neurons quickly returned to normal circadian rhythms, and osteoporosis gradually recovered (Lucassen et al., 2016). 4-week-old rats were exposed to a 12 h light (8:00–20:00) and 12 h dark (20:00–8:00) photoperiods for 4 weeks. One group was fed 4 hours after the beginning of light, and the other group fed 4 hours after the beginning of dark. The authors observed that different eating times disturbed the normal rhythms of DNA and collagen synthesis in rat tibias (Russell et al., 1983). The relationship between food intake and bone mass in 2-month-old rats was investigated, and a peak in bone resorption was observed after eating, however, dividing food into smaller pieces weakened this effect. However, the effect was not weakened in parathyroidectomy rats, suggesting calcitonin inhibited bone resorption, as mediated by osteoclasts. (Muhlbauer and Fleisch, 1995). Phosphate was an important metabolite in regulating circadian rhythm functions in bone tissue and peripheral non-bone tissue, *via* low phosphorus feeding in male 8–10 weeks fracture old mice (Noguchi et al., 2018) (Table 5).

**TABLE 5:** Biological rhythm disorders in bone tissue lead to osteoporosis in animal studies.

Animal	Interference	Consequence		Reference
Mice	24 hour light	Trabecular density	↓	Luccasen et al. (2016)
Rats	12:00 fed	DNA synthesis20:00+		Russel et al. (1983)
		Collagen synthesis08:00+		
	24:00 fed	DNA synthesis16:00+		
		Collagen synthesis12:00+		
Rats	Eating	Bone resorption		Muhlbauer & Fleisch. (1995)
Fracture mice	Low phosphorous feeding	Bone volume	↓	Noguchi et al. (2018)

-: nadir,+:zenith

### 6.3 Human studies

6,510 women (≥40 years old) were studied and when total sleep time is fixed, delayed sleep or insufficient sleep at night, led to increased bone loss in postmenopausal, but not in premenopausal women (Wang et al., 2015). Circadian rhythm interference studies were performed on six healthy 20–27 year old men, and four healthy 55–65 year old men for 3 weeks (sleep times were set at 5–6 h/day). After interference, serum P1NP levels decreased, while CTX levels remained unchanged, suggesting an imbalance in bone turnover, i.e., bone formation was reduced but bone resorption was unchanged. Thus, circadian rhythm disorder and sleep deprivation may be influential factors for bone damage (Swanson C. M. et al., 2017). The relationship between resting activity circadian rhythm patterns and BMD were monitored by following-up 5,994 elderly men (≥65 years old). Using a generalized linear model, the circadian activity ratio was related to hip joint and femoral neck BMD, but it was not an independent influential factors (Rogers et al., 2017). 96 nighttime and 100 daytime workers were studied, and night work caused vitamin D deficiency, potentially indicative for osteoporosis (Romano et al., 2015). A randomized crossover study on 11 healthy premenopausal women (24 ± 5 years old) was performed to assess circadian rhythm changes on bone turnover markers in subjects consisting of two periods: either 33 h of fasting (fasting) followed 1 week later by a 33-h period with regular meals eaten at 08:00–08:30,11:30–12:30 and 18:00–19:00 (control) or *vice versa*. Food intake had little effect on the diurnal changes of bone resorption, and was independent of PTH levels. Decreased iPTH (intact PTH) during fasting may be secondary to increased bone resorption caused by fasting (Schlemmer & Hassager, 1999). Serum CTX, a marker of bone resorption after fasting, oral glucose tolerance tests (OGTT) and normal eating were detected in postmenopausal women, premenopausal women and men. The circadian rhythm of bone resorption was mediated by eating and fasting, and independent with gender and menstrual status. The circadian rhythm of bone resorption suggests it decreases when eating during the day, and increases when fasting at night (Bjarnason et al., 2002). An 8-years follow-up study was conducted on 38,062 postmenopausal women and when compared with women who never worked a night shift, those with 20 years night work experience had an increased risk of hip and wrist fractures (Feskanich et al., 2009). 70 postmenopausal women (39 on night and 31 on day shift) were studied, and the BMD of trabecular and cortical bones of night shift workers was lower, suggesting night shift work altered circadian rhythms, and was a risk factor for osteoporosis (Quevedo & Zuniga, 2010). Cortisol secretion from 10 healthy postmenopausal women was intervened to study circadian rhythm etiology in bone. Subjects were divided into two groups. Firstly, endogenous cortisol secretion of both groups was blocked by metoprolone. Then, one group had different hydrocortisone doses according to the circadian rhythm at each time point, while the other group had the same hydrocortisone dose at each time point to eliminate the circadian rhythm. Serum OC levels changed in line with the circadian rhythm of serum cortisol, but procollagen type I carboxyl peptide (PICP) and bone resorption markers did not. This study showed that the circadian rhythm of bone resorption was not mediated by cortisol (Heshmati et al., 1998). A similar conclusion by studying 35 women (11 patients with hypopituitarism and 24 healthy women) was reached. In patients without circadian rhythm of cortisol, there were normal circadian rhythm changes of pyridine cross-linking compounds and PICP in urine, but no circadian rhythm changes of serum OC. These observations suggested that circadian changes in serum OC may have been mediated by endogenous circadian changes in serum cortisol, but serum cortisol could not control circadian changes in serum PICP, nor the rhythmic changes of pyridine cross-linking compounds in urine (Schlemmer et al., 1997) (Table 6).

**TABLE 6:** Biological rhythm disorders in bone tissue lead to osteoporosis in human studies.

Human	Interference	Consequence		Reference
6510 women(≥40 years old)	Delayed sleep or insufficient sleep	Bone loss	↑	Wang et al. (2015)
6 men (20-27 years old),and 4 men(55-65 years old)	Sleep times 5-6 hours/day	Bone formation	↓	Swanson et al. (2017b)
5994 men(≥65 years old)	Weaker RAR patterns	BMD	↓	Rogers et al. (2017)
96 nighttime workers	VitaminD deficiency	Osteoporosis		Romano et al. (2015)
11 women(24±5 years old)	Fasting	Bone resorption	↑	Schlemmer & Hassager. (1999)
14 postmenopausal women, 23 premenopausal women and men	Fasting	Bone resorption	↑	Bjarnason et al. (2002)
	Eating	Bone resorption	↓	
38062 postmenopausal women	Night work	Hip and wrist fractures	↑	Feskanich et al. (2009)
70 postmenopausal women	Night shift workers	BMD of trabecular and cortical bones	↓	Quevedo & Zuninga. (2010)
10 postmenopausal women	Eliminate the cortisol rhythm	OC rhythm changed		Heshmati et al. (1998)
11 patients with hypopituitarism and 24 healthy women	Without circadian rhythm of cortisol	No circadian rhythm of serum OC		Schlemmer et al. (1997)

↑: increase, ↓: decrease

## 7 The application of bone tissue rhythms

81 postmenopausal women were studied and the oral administration of 0.8 mg salmon calcitonin at three different time points (8:00, 17:00, and 22:00) reduced CTX serum levels, i.e., bone resorption was inhibited. In addition, different administration times had different effects on bone resorption i.e., taking the supplement before dinner exerted the best effects, with a bone resorption inhibition rate of 25% (Karsdal et al., 2008). 50 postmenopausal women with osteoporosis were investigated, after 12 months of treatment with tripatide (TPTD), lumbar BMD in the morning treatment group was higher than in the night treatment group. This showed that TPTD administration time had a significant impact on osteoporosis (Michalska et al., 2012). Pulsed electromagnetic fields (PEMF) can prevent osteoporosis, and was applied to 3-month-old ovariectomized female rats at different time points, and observed that the therapeutic effects from 9:00–15:00 were significantly improved when compared to the 0:00–6:00 period (Jing et al., 2010).

## 8 Future perspectives

Melatonin, as a therapy for osteoporosis, has been investigated by several pivotal studies, and valued by researchers and clinicians. Melatonin appears to adjust and restore circadian rhythms, deepen sleep, improve both sleep quality and the functional state of the body. There are biorhythms related proteins in bone tissue. Therefore, melatonin promotes bone formation and inhibits bone resorption. This latter mechanism is facilitated by the differentiation and maturation of osteoclasts, which is partly mediated by rhythmic proteins, therefore, melatonin as an osteoporosis therapeutic is feasible.

By consulting the literature and databases, we found no preclinical reports on melatonin or rhythmic protein as therapeutic targets for osteoporosis. Therefore, in the future, we must focus on how melatonin maintains normal bone biological rhythms *via* rhythm proteins.

By applying new technologies and performing more comprehensive human and animal studies, new biological rhythm mechanisms will be identified. These approaches will provide greater insights on biological rhythms, and contribute to our knowledge on osteoporosis. We hope in the future, melatonin will be incorporated into clinical practice as an osteoporosis therapy, and provide hope and relief for patients with the condition.
